# Success Rate of Wire Control-Assisted ERCP Sphincterotomy Versus Non-assisted ERCP Cannulation of Common Bile Duct in a Secondary Care Unit During the First COVID-19 Peak: A Retrospective Observational Study of 281 Patients

**DOI:** 10.7759/cureus.17861

**Published:** 2021-09-09

**Authors:** Eyad Gadour, Okwudili Agu, Mutwakil Musharaf, Megan Dixon, Amr Askar, Siddrah Hafeez, Yousuf Shafiq, Abdalla Arabiyat, Julia Moradi

**Affiliations:** 1 Gastroenterology and Hepatology, University Hospitals of Morecambe Bay NHS Foundation Trust, Lancaster, GBR

**Keywords:** endoscopy ercp, covid 19, common bile duct (cbd), sphincterotomy, wire control-assisted ercp, non-assisted ercp

## Abstract

Background

The British Society of Gastroenterology (BSG) recommended that during the COVID-19 pandemic, endoscopy units perform endoscopic retrograde cholangiopancreatography (ERCP) for obstructive biliary pathologies in an emergency. We assessed the local performance of ERCP during the first wave of COVID-19 at our local endoscopy center, in particular the technique to common bile duct (CBD) cannulation.

Methodology

All ERCP procedures performed from January to June 2020 were retrospectively assessed and compared with procedures performed between January and June 2019 at the Royal Lancaster Infirmary. The indications for ERCP, success rate, and complications were studied separately. Correlation analysis was conducted using Spearman's rank correlation coefficient. The binary logistic regression model was used to compute the factors associated with successful ERCP. Significance was established when the two-sided P-value < 0.05. Statistical analysis was performed using Statistical Package for the Social Sciences (SPSS) software version 25 for Windows (SPSS Inc., Chicago, IL, USA, 2017).

Results

A total of 281 ERCP were included in this study, with 169 and 112 performed during the first six months of 2019 and 2020, respectively. A statistically significant (0.0087) higher proportion of cases with liver dysfunction presented for ERCP before the COVID-19 outbreak (152, 89.94%). All patients before COVID-19 underwent wire control-assisted ERCP, while 82 (73.21%) received assisted ERCP during the first wave (P < 0.001). There was no statistically significant difference (P = 0.10) in the number of patients who underwent sphincterotomy before and during the first wave of COVID-19, with 97 (57.39%) and 76 (67.85%), respectively. The success rate of ERCP before COVID-19 was relatively high, accounting for 146 (86.39%) patients in contrast to 87 (77.67%) patients during the first wave (P = 0.074). Sphincterotomy (*β* = 2.800, P = 0.028) and stent insertion (*β* = 0.852, P = 0.046) were statistically significant predictors of ERCP outcomes. There was no statistically significant impact of cholangitis on the success of ERCP (*β* = 1.672, P = 0.109).

Conclusion

The first wave of COVID-19 had a statistically proven negative impact on the expected standards of ERCP performance. Although the complication rate was significantly higher during the first wave case difficulty, the American Society of Anesthesia (ASA) status was not assessed on an individual basis. Both ASA status and case difficulty are now included in our endoscopy selection process. We recommend adding the complexity of cases and ASA to the local and national recording databases. This is a rare study on UK-based hospitals.

## Introduction

In December 2019, a new strain of the coronavirus family, severe acute respiratory syndrome coronavirus 2 (SARS-CoV-2), was isolated for the first time from the respiratory tract of many patients suffering from pneumonia in Wuhan, China [1]. In March 2020, the World Health Organization (WHO) named SARS-CoV-2 infection COVID-19 as it had spread to around 114 countries by March 2020. Patients showed a clinical presentation of respiratory illness with fever, shortness of breath, dry cough, and oral/fecal transmission also being part of this clinical picture [2]. Such transmission features of the coronavirus, which is airborne and direct contact, put endoscopy departments at a high risk of being affected by the respiratory disease during endoscopic procedures involving oral and fecal aspiration [3]. At the beginning of this outbreak in Wuhan, 29% of patients were healthcare workers (HCWs) who were infected by patients carrying this novel strain of COVID-19 [4]. Subject to the route of spread of coronavirus, the use of personal protective equipment (PPE) has been strongly recommended for clinical procedures in medical societies to avoid its spread [3].

Endoscopic retrograde cholangiopancreatography (ERCP) is a technique used for the management of various pancreatic, biliary duct, and ampulla, the confluence of both ducts, disorders and is generally regarded as efficacious and safe with documented benefits in comparison to surgical approaches [5]. In addition to the management of these disorders, ERCP can be implemented as a therapeutic intervention [6]. This technique involves the use of a specially designed endoscope - a long, flexible tube with an installed camera on its top that passes through the gastrointestinal tract to the duodenum. The installed camera assists the gastroenterologist by showing a magnified view of the gastrointestinal tract. To obtain x-rays, the radiocontrast agent is injected into the pancreatic or bile duct via a tube. In addition, various specialized equipment is passed through an open channel of this endoscope for different clinical procedures such as biopsy, dilation of vessels, placement of a stent, or retrieving stones [6]. Following this procedure can also aid in treating various diseases of the liver, pancreas, and gallbladders such as diagnosis and treatment of blockage of the bile duct due to cancer and gallstones, jaundice, persistent pain in the gastrointestinal tract that cannot be diagnosed with other techniques, and cancer of the gallbladder or bile duct [6]. In addition to the beneficial outcomes of ERCP procedures, various complications can also occur after the procedure, such as hemorrhage, bleeding, pancreatitis, and infection. Cholangitis is the most serious complication, which may result in septicemia [5]. Common organisms responsible for infection followed by ERCP include Enterobacteriaceae, Pseudomonas, *Pseudomonas*​​​​* aeruginosa*, and *Staphylococcus epidermidis* [7]. The risk of post-ERCP infection is increased in cases where percutaneous and endoscopic procedures are performed simultaneously in immunocompromised patients, in the presence of jaundice, and when there is no or incomplete drainage of biliary fluids [8].

Workers in endoscopic departments, including gastroenterologists, are more prone to being exposed to respiratory and gastrointestinal fluids during clinical procedures as the probability of transmission of coronavirus is greater during the endoscopic procedure [9]. Endoscopic departments were acutely affected worldwide, and this pandemic has led to the stoppage of elective and endoscopic procedures globally [10]. Many international endoscopy organizations such as the European Society of Gastrointestinal Endoscopy [11], the American Society for Gastrointestinal Endoscopy, and the World Endoscopy Organization [3,12] have emphasized endoscopic procedures. These organizations issued guidelines for endoscopic procedures such as the use of PPEs, negative pressure rooms, and viral testing for COVID-19, which further led to an increase in the cost of endoscopic procedures and a decrease in the total number of procedures [13]. These guidelines also suggested deferring minor cases while considering emergency cases only to halt the risk of spread of coronavirus in HCWs. All such protective measures led to a significant decrease in endoscopic procedures as a significant decrease was observed in the detection rate of gastrointestinal cancer [14]. The emergence of asymptomatic patients carrying the virus and transmitting it on close contact poses further challenges for endoscopic procedures [15].

On January 31, 2020, the first patient with a positive COVID-19 test in the United Kingdom was confirmed [16]. In mid-March 2020, the pandemic reached its peak leading to a lockdown in the United Kingdom [17]. To deal with the pandemic, hospitals in the United Kingdom restructured their services, and guidelines were issued by the British Society of Gastroenterology (BSG) to reduce the endoscopic procedures and to consider emergency endoscopic procedures only [18].

This study evaluated the impact of the COVID-19 pandemic on ERCP performance at Royal Lancaster Infirmary during the first wave of the pandemic in the United Kingdom.

## Materials and methods

Retrospectively, a cohort study was performed to review the clinical notes and procedures reports of all ERCP databases in our secondary endoscopic unit at Royal Lancaster Infirmary in North-West of the United Kingdom. All ERCP performed for adults were divided into two main groups depending on the time of presentation: Group 1, before COVID-19 from January 2019 to June 2019, and Group 2, during the first COVID-19 wave to the plateau from January 2020 to June 2020. Demographic data, indications of the procedure, radiological tests, types of intervention, and complications data were collected for the above-mentioned periods and compared. Informal consent was waived as this was only a retrospective data analysis.

The two main categorical classifications of the successful CBD cannulation in the study were divided into two: (1) wire control assisted, where the endoscopist assistant controls the guidewire in the process of cannulation versus (2) the other technique, non-assisted, where the endoscopist would independently control both the scope and the guidewire. 

Statistical analysis

Categorical variables were expressed as numbers and percentages, and their particular groups were compared using Pearson’s chi-square test with Fisher’s exact test. Non-normally distributed data were reported using median and range, and its related groups were compared using the Kruskal-Wallis test. Correlation analysis was conducted using Spearman's rank correlation coefficient. A binary logistic regression model was used to compute the factors associated with successful ERCP. Significance was established when the two-sided P-value < 0.05. Statistical analysis was performed using Statistical Package for the Social Sciences (SPSS) software version 25 for Windows (SPSS Inc., Chicago, IL, USA, 2017). The figures were rendered using GraphPad Prism software version 8 (GraphPad Software Inc., San Diego, CA, USA).

## Results

Demographic characteristics of the patients

**Figure 1:**
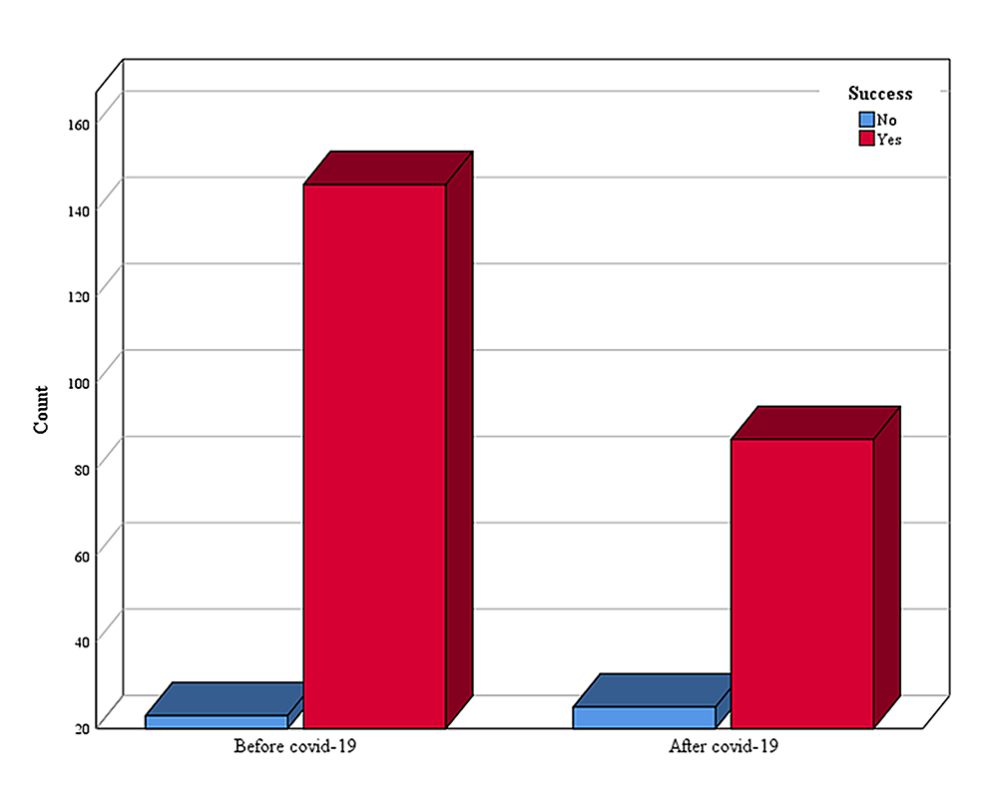
Proportion of patients with successful and failed ERCP before and during the first wave of COVID-19 ERCP, Endoscopic retrograde cholangiopancreatography.

This study included 281 patients presented for ERCP. Of these, 169 (60.14%) presented before the COVID-19 era compared to 112 (39.86%) who presented during the first wave. The median age of the patients before COVID-19 was 76 (27-101) and 73 (21-95) years after COVID-19 (P = 0.079). Furthermore, 90 (53.35%) females were subjected to ERCP before COVID-19, and 55 (49.10%) presented during the first wave (P = 0.0542). Figure 1 shows the proportion of patients with successful and failed ERCP before and during the first wave of COVID-19.

**Figure 2:**
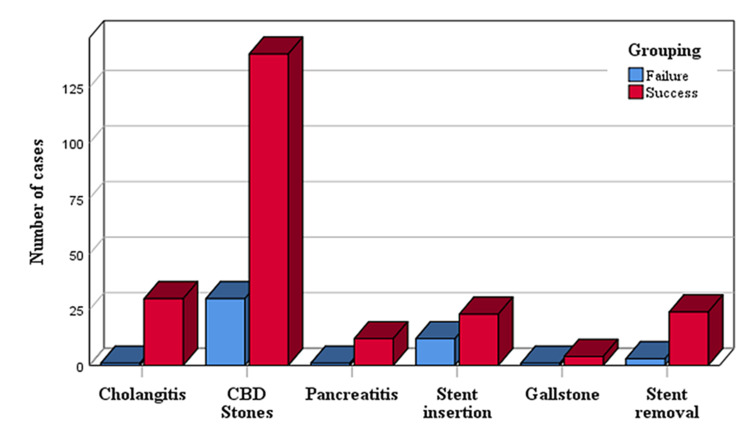
Proportion of patients with successful and failed ERCP stratified by the indication of ERCP ERCP, Endoscopic retrograde cholangiopancreatography; CBD, common bile duct.

A statistically significant (0.0087) higher proportion of cases with liver dysfunction presented for ERCP before the COVID-19 outbreak, accounting for 152 (89.94%) patients. In contrast, there was a statistically significant (P = 0.0036) higher proportion of patients presented with a history of the previous ERCP during the first wave, accounting for 84 (75%) patients compared to 83 (49.11%) presented before the outbreak. Regarding ERCP indications, there was no statistically significant difference between patients presented before and during the first wave (P = 0.414). The administration of prophylactic antibiotics was statistically significantly higher (P < 0.001) among patients presented during the first wave compared to those presented before it, with 81 (72.32%) and 94 (55.62%) patients, respectively (Table 1). The proportion of patients with successful and failed ERCP stratified by the indication of ERCP is presented in Figure 2.

**Table 1:** Baseline demographic characteristics of studied patients before and during the first COVID-19 wave ERCP, Endoscopic retrograde cholangiopancreatography; CBD, common bile duct.

Variables	Before COVID-19	During COVID-19	P-Value
	Number (%)/Median (Range)	Number (%)/Median (Range)	
Number	169	112	
Age (Years)	76 (27-101)	73 (21-95)	0.079
Gender			
Females	90 (53.35%)	55 (49.10%)	0.054
Males	79 (46.74%)	57 (50.89%)	
Liver dysfunction	152 (89.94%)	89 (78.76%)	0.0087
Previous ERCP	83 (49.11%)	84 (75%)	0.0036
Indication for ERCP			0.414
Cholangitis	15 (8.87%)	16 (14.28%)	0.176
CBD stones	101 (59.76%)	69 (61.60%)	0.803
Pancreatitis	8 (4.73%)	5 (4.46%)	
Stent insertion	21 (12.42%)	14 (12.5%)	
Gallstones	4 (2.3650%)	1 (0.89%)	0.0651
Stent removal	20 (11.83%)	7 (6.25%)	0.149
Prophylactic antibiotic	94 (55.62%)	81 (72.32%)	<0.001

ERCP-related outcomes

Before the COVID-19 era, two (1.18%) patients underwent ERCP within 72 hours, in contrast to five (4.46%) during the first wave of COVID-19 (P = 0.231). All patients before COVID-19 underwent wire control-assisted ERCP, while 82 (73.21%) underwent assisted ERCP during the first wave (P < 0.001). There was no statistically significant difference (P = 0.10) in the number of patients who underwent sphincterotomy before and during the first wave with 97 (57.39%) and 76 (67.85%), respectively. The success rate of ERCP before COVID-19 was relatively high, accounting for 146 (86.39%) patients in contrast to 87 (77.67%) during the first wave (P = 0.074). During the first wave of COVID-19, there was a statistically significant trend in the administration of non-steroidal anti-inflammatory drugs (NSAIDs) post-ERCP with 101 (90.17%) patients to 141 (83.43%) before the COVID-19 (P = 0.019). There was a higher risk of post-ERCP complications among patients who underwent ERCP with a complication rate of 22.33% compared to 3.56% before COVID-19 (P = 0.019) (Table 2).

**Table 2:** Comparison between wire control-assisted and unassisted ERCP success and complication review ERCP, Endoscopic retrograde cholangiopancreatography.

Variables	Before COVID-19	During the First Wave	P-Value
	Number (%)	Number (%)	
Number	169	112	
ERCP within 72 h	2 (1.18%)	5 (4.46%)	0.231
Wire control			
Wire control-assisted	169 (100%)	82 (73.21%)	<0.001
Wire unassisted	0 (0%)	30 (26.78%)	
Sphincterotomy	97 (57.39%)	76 (67.85%)	0.10
Analgesics	141 (83.43%)	101 (90.17%)	0.019
Success of ERCP	146 (86.39%)	87 (77.67%)	0.074
Complications of ERCP			
None	163 (96.44%)	100 (77.67%)	0.019
Bleeding	6 (3.55%)	6 (5.35%)	
Death	0 (0%)	1 (0.89%)	
Pancreatitis	0 (0%)	5 (4.46%)	

Factors associated with ERCP outcomes

There was a statistically significant negative association between cholangitis and ERCP success (r = -0.129, P = 0.030). In this respect, stent insertion (r = -0.172, P = 0.004) showed a statistically significant negative association with successful ERCP. In contrast, sphincterotomy (r = 0.232, P < 0.001) showed a statistically significant positive correlation with successful ERCP. There was no statistically significant association between successful ERCP and patient age (P = 0.116), ERCP within 72 hours (P = 0.463), and methods of wire control (P = 0.110) (Table 3).

**Table 3:** Factors associated with ERCP outcomes ERCP, Endoscopic retrograde cholangiopancreatography; CBD, common bile duct.

Variables	Correlation Coefficient (r)	P-Value
Age	-0.103	0.116
Gender	-0.033	0.576
Liver dysfunction	-0.056	0.352
Previous ERCP	0.023	0.696
Indication of ERCP		
Cholangitis	-0.129	0.030*
CBD Stones	-0.018	0.756
Pancreatitis	0.054	0.359
Stent insertion	0.172	0.004*
Gallstones	-0.010	0.862
Stent removal	0.032	0.587
Prophylactic antibiotic	-0.021	0.725
ERCP within 72 h	-0.044	0.463
Wire control	0.096	0.110
Sphincterotomy	0.232	<0.001*
Analgesics	-0.013	0.831

**Figure 3:**
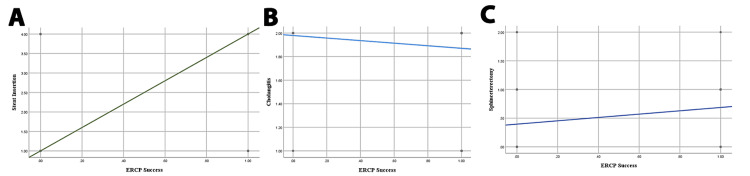
Scatter plots with regression lines showing the association between success of ERCP and (A) stent insertion, (B) cholangitis, and (C) sphincterotomy. ERCP, Endoscopic retrograde cholangiopancreatography.

In binary logistic regression analysis, sphincterotomy (β = 2.800, P = 0.028) and stent insertion (β = 0.852, P = 0.046) were statistically significant predictors of ERCP outcomes. There was no statistically significant impact of cholangitis on the success of ERCP (β = 1.672, P = 0.109) (Figure 3, Table 4).

**Table 4:** Binary logistic regression model for factors associated with ERCP outcomes ERCP, Endoscopic retrograde cholangiopancreatography.

Variables	β	Standard Error	P-Value
Sphincterotomy	2.800	1.274	0.028
Cholangitis	1.672	1.044	0.109
Stent insertion	0.852	0.426	0.046

## Discussion

ERCP and sphincterotomy are both broadly recognized as first-line options for the removal of stones in bile and pancreatic ducts. Similarly, performing an ERCP procedure within three days lessens the risk of suffering from acute pancreatitis and further associated complications [19]. As the ERCP procedure is an aerosol-generating procedure, it puts workers at a higher risk of suffering from COVID-19. Additionally, due to the emergence of asymptomatic patients, the transmission of the virus from these patients, and the appearance of many false-negative tests for COVID-19, it becomes challenging to perform screening of all COVID-19 patients before performing ERCP [20]. Subject to this increased risk, various guidelines have been issued for carrying out these procedures, and international endoscopy societies recommend urgent ERCP for emergency cases such as acute obstruction of the bile duct, which requires stent installation and acute cholangitis [21].

We analyzed the impact of the pandemic on the ERCP procedure in our center by comparing the data of two periods, that is before and during the first wave of the COVID-19 pandemic. A six-month period was evaluated with respect to ERCP services. A lower number of patients were presented for ERCP during the pandemic period (39.86%) compared to the period before the pandemic (69.14%). These results were consistent with other regions across the globe where a similar significant decrease in the number of ERCP procedures was observed, such as in the United States, China, the Netherlands, and Romania [22-25]. Similarly, Rutter et al. also reported a 44% reduction in ERCP services in the United Kingdom [14]. Reduction in such services in the United Kingdom was initially observed during mid-March 2020 when the pandemic was at its peak, and healthcare facilities restructured their services to control the pandemic and thus restricted endoscopic services. The BSG issued national guidelines for endoscopy services on March 23, 2020, to stop non-emergency endoscopic services [18].

The number of cases with liver dysfunction was surprisingly lower during the pandemic period than during the non-pandemic period (P < 0.05). Different organizations have proposed guidelines for the management of liver dysfunction during endoscopic procedures during the pandemic including the use of PPEs and postponing of certain procedures as, during the pandemic, liver dysfunction could be of varying degrees and complications, which may result in immune response and systemic inflammation and may also alter drug pharmacokinetics [26].

The outcomes revealed some statistically insignificant differences indicating a comparative increase in ERCP indications for cholangitis, common bile duct stones, and stent insertion of 88.7%, 59.76%, and 12.4%, respectively, before the COVID-19 period and 14.28%, 61.60%, and 12.5% after the COVID-19 period, respectively. The success rate of ERCP procedures was found to be relatively higher before the pandemic (86.39%) than that observed during the pandemic (77.67%). Similar outcomes have been reported around the world [24,27,28]. This reduction in success rate can be attributed to various factors, such as restrictive guidelines issued by international and national organizations that endorsed the use of PPEs and also recommended the reduction of staff in endoscopy departments. Another factor may be the reduction in the number of training programs or their complete stoppage, which could be a reason for the reduction in the success rate of ERCP procedures. Forbes et al. compared 73 departments in North America and reported that 49% had reduced endoscopy training and 45% of these completely stopped such programs [22]. Similarly, an international survey involving more than 60 countries reported that in more than 93% of cases of endoscopy, the involvement of trainees was halted, and the same study also reported a shortage of PPE in 28.8% of cases from a total of 770 participants [29].

Post-ERCP complications were observed to be significantly higher than those before the pandemic. A complication rate of 22.33% was observed during the COVID-19 period compared to 3.5% before the COVID-19 period (P-value = 0.019). Bleeding and pancreatitis were the two major complications observed during the COVID-19 period, and death occurred in one case. A complication of bleeding was slightly higher, but the occurrence of pancreatitis was not observed before the pandemic and was observed in 5.35% of cases during the pandemic (P < 0.05). Post-ERCP pancreatitis has been reported as a specific complication of endoscopy [30]. Thus, the performance of endoscopic procedures during a pandemic requires specific decisions for the management of such complications. There is an increased risk of mortality during the performance of endoscopic procedures, especially during upper gastrointestinal endoscopy procedures in a COVID-19-positive patient, and increased bleeding complications may be attributed to coagulopathy associated with the virus; for its prevention and management, close monitoring of prothrombin time and platelet count are suggested [26].

Correlation analysis was conducted to evaluate the relationship between various factors and the success of the ERCP procedure for all patients, that is patients in both the pre- and post-pandemic periods. We observed a statistically significant but negative correlation between cholangitis and stent insertion and success of ERCP (r = -0.129) and (r = -0.172), respectively, for both of these complications (P < 0.05). On the other hand, we observed a strong positive and statistically significant correlation between sphincterotomy and the success of ERCP (r = 0.232, P < 0.001). There was no correlation between the success of ERCP and the age of the patient and the ERCP procedure within three days. A systemic review compared four studies in which the ERCP procedure was performed within and after 72 hours, but no significant reduction in mortality rate was observed with respect to the odds ratio (OR = 0.32, 95% CI 0.15-0.68). Binary logistic regression analysis revealed a significant relationship between sphincterotomy and stent insertion with respect to outcomes of ERCP with a value coefficient of β of 2.800 and 0.852, respectively, (P < 0.05) and an insignificant relationship between cholangitis and outcomes of ERCP (β = 1672, P > 0.05).

The overall reduction in the endoscopic procedures including ERCP after the emergence of the pandemic resulted in a marked decrease in cancer detection worldwide as the absolute number reduced dramatically due to COVID-19. This decrease in endoscopic procedures, including ERCP, occurred due to strict guidelines for these procedures to be carried out during the pandemic [14]. Some organizations and healthcare facilities also endorsed the stoppage or reduction of endoscopic procedures to reserve all available sources to fight the pandemic, to ensure the safety of medical and paramedical staff in healthcare facilities, and to utilize a maximum of the staff to deal with the pandemic [31]. Contrary to these findings, some studies also reported that there was no significant decrease in ERCP services during the pandemic because most procedures were carried out in emergency situations [20]. Another factor in not postponing these procedures was that the duration of the pandemic was unknown [27,32,33]. Similarly, a study reported the safety of ERCP procedures where ERCP was conducted for 18 patients who tested positive for COVID-19. The study compared COVID-19 and a control group with respect to complications of the ERCP procedure and found no significant impact on patient safety [34].

Most endoscopic departments have resumed endoscopic services, including ERCP, which were restricted due to COVID-19. The resumption of such services was led by international societies after issuing guidelines for these procedures to compete with the global health crisis. These guidelines included in-depth preparation of endoscopy staff and patients, use of PPE, and constant staff [18,35].

## Conclusions

The COVID-19 outbreak has had a significant influence on the overall endoscopic services across the world, including ERCP services, which resulted in a persistent reduction in the total number of ERCP procedures worldwide, especially in the United Kingdom. In addition to the number of procedures, the success rate of ERCP procedures was significantly reduced. Complications related to ERCP services could occur even after adhering to proper guidelines and with increased probability during the COVID-19 pandemic. Our results reveal that the impact of COVID-19 will lead to a reduction in the detection of cancers and other disorders of the bile duct, pancreatic duct, and ampulla, leading to increased morbidity and mortality related to these complications. These services could be safely resumed without compromising the health and safety of both patients and endoscopists by adopting and following various Standard of Procedure (SOP) and guidelines issued by international organizations, such as the use of PPEs, limiting the number of personnel, through the preparation of staff and patients before ERCP, regular testing for COVID-19, and so on. The study suggests that organizations should take significant steps to resume endoscopic and ERCP services.
